# How Early Should I Refer My Patient? The Benefits of a Quick Ophthalmic Referral in Spinocerebellar Ataxias, a Case Series and Literature Review

**DOI:** 10.3390/brainsci16070756

**Published:** 2026-07-17

**Authors:** Andrea B. Fiscal-Carvajal, José L. De-León-Guerra, Cristian E. Salinas-Aguirre, Marisol Ibarra-Ramírez, Marissa L. Fernández-de-Luna, Ingrid E. Estrada-Bellmann, Joel Arenas-Estala, Luis D. Campos-Acevedo, Jibran Mohamed-Noriega

**Affiliations:** 1 Department of Genetics, University Hospital and Faculty of Medicine, Autonomous University of Nuevo Leon (UANL), Monterrey 64460, Mexico; andrea.fiscalc@uanl.edu.mx (A.B.F.-C.); mibarrar@uanl.edu.mx (M.I.-R.); joel.arenase@uanl.edu.mx (J.A.-E.); lcamposa@uanl.edu.mx (L.D.C.-A.); 2 Department of Ophthalmology, University Hospital and Faculty of Medicine, Autonomous University of Nuevo Leon (UANL), Monterrey 64460, Mexico; luis.deleongrr@uanl.edu.mx (J.L.D.-L.-G.); cristian.salinasagrr@uanl.edu.mx (C.E.S.-A.); mfernandez.109881@uanl.edu.mx (M.L.F.-d.-L.); 3 Department of Neurology, University Hospital and Faculty of Medicine, Autonomous University of Nuevo Leon (UANL), Monterrey 64460, Mexico; ingrid.estradabm@uanl.edu.mx

**Keywords:** spinocerebellar ataxias, trinucleotide repeat expansion, ophthalmological diagnostic technique, genetic testing, rare diseases

## Abstract

**Highlights:**

**What are the main findings?**

- Early ophthalmologic assessment helped narrow the differential diagnosis and facilitated molecular confirmation of SCA2, SCA7, and SCAR32 in patients with prolonged diagnostic delays.
- Five key clinical features—macular abnormalities, optic nerve involvement, hearing loss, erythrokeratodermia, and dementia—can help classify spinocerebellar ataxias and guide targeted genetic testing.

**What are the implications of the main findings?**

- Prompt multidisciplinary referral involving ophthalmology, neurology, and clinical genetics may reduce diagnostic time and improve the diagnostic workup of patients with suspected spinocerebellar ataxia.
- Recognition of key clinical features can support a more efficient selection of molecular tests, reduce unnecessary investigations, and improve genetic counseling for affected families.

**Abstract:**

**Background**: The spinocerebellar ataxias (SCA) are a group of multiple inherited disorders. The molecular diagnosis of the specific type of SCA is challenging due to the diversity of hereditary patterns and genetic anomalies. Certain key clinical features can help shorten the differential diagnosis. **Cases**: At our center, we evaluated three patients presenting with gait disturbances. They were assessed by multiple hospital departments, and, due to suspected spinocerebellar ataxia, comprehensive genetic and ophthalmologic testing was performed, leading to diagnoses of SCA2, SCA7, and SCAR32. These cases highlight the importance of multidisciplinary evaluation for accurate diagnosis of complex neurogenetic disorders. All patients experienced prolonged diagnostic delays, requiring years of multiple consultations across different hospitals before reaching a definitive diagnosis. **Literature Review**: Among all published reports of patients with SCA, we consider that five distinct key clinical features can categorize many patients suffering from a possible SCA into different groups and reduce the potential differential diagnosis. Only two types of SCA presented macular anomalies, ten optic nerve involvements, one erythrokeratodermia, four hearing losses, and four dementias. **Conclusions**: Early referral to a multidisciplinary evaluation might help narrow the differential diagnosis of specific types of SCA, guiding the diagnostic work-up toward SCA1 and SCA7 in patients with macular involvement, or toward SCA1, SCA2, SCA3, SCA7, SCAR3, SCAR9, SCAR21, SCAR29, SCAR31, and SCAX3 in those with optic nerve involvement.

## 1. Introduction

Spinocerebellar ataxias (SCA) comprise a group of disorders that exhibit similar symptoms but differ in their genetic causes and accompanying clinical findings. Most SCAs are hereditary, but the inheritance pattern can be Autosomal Dominant (AD), Autosomal Recessive (AR), or X-linked trait. An increased number of repeated trinucleotide CAG causes many of the AD SCAs. These trinucleotide expansions lead to the production of proteins that, upon translation, contain abnormally long polyglutamine tracts. The expansions of these trinucleotides are progressive across generations, and there is a direct correlation between the number of repeats and both the severity and age of onset of the disease, a phenomenon called anticipation [1].

However, not all SCAs are caused by a polyglutamine expansion; others are caused by pentanucleotides (SCA10, SCA31), hexanucleotides (SCA36), or even point variants (SCA13, SCA14, SCA18) [2]. Spinocerebellar ataxias that are AR (SCAR) are often related to mutations affecting mitochondrial metabolism, DNA repair, or antioxidant homeostasis [3]. In X-linked spinocerebellar ataxias (SCAX), the involvement of various genes, such as *GJB1, ABCD1*, and *WDR45,* leads to alterations in intercellular communication, fatty acid transport, and cellular autophagic processes, respectively, resulting in progressive neuronal degeneration [4].

Due to the different types of genetic anomalies that cause SCA, the diagnosis can be challenging and often delayed. For instance, the average time to diagnose a rare disease is around 6 years [5]. SCA as a group is not typically the first condition that comes to mind when facing a patient with abnormal movements. However, it is crucial to consider and address it by a team of specialists, including neurologists, ophthalmologists, radiologists, and geneticists. This multidisciplinary approach can lead to a quicker diagnosis, more effective treatment, and an improved prognosis for patients.

The prompt identification of accompanying clinical findings can accelerate the timely diagnosis of SCA, reduce the differential diagnosis, and help clinicians target diagnostic tests to a specific type or group of SCA. Ophthalmic symptoms have been reported in most types of SCA; however, most patients with SCA only report abnormal ocular movements, and only a few kinds of SCA will develop retinal or optic nerve abnormalities. It is important that the ophthalmologic evaluation include multimodal imaging [6].

The present report highlights the importance of early ophthalmic and genetic referral in narrowing the possible SCAs associated with anomalies and reviews the literature to categorize accompanying clinical characteristics into five groups of key clinical features.

## 2. Case Series

### 2.1. Case 1

**Figure 1:**
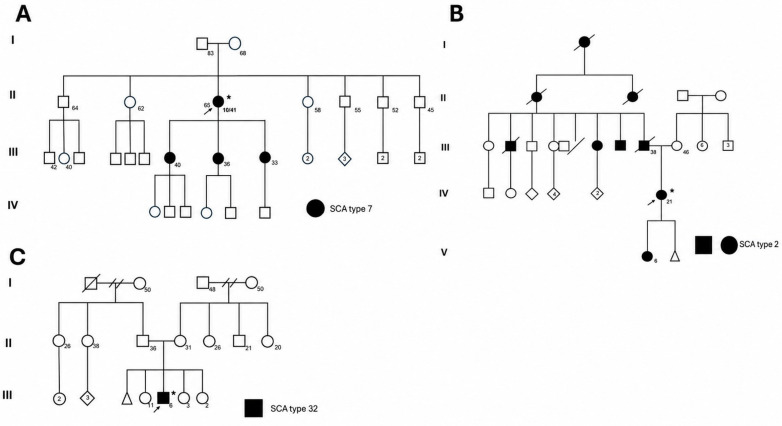
Pedigree of three families with SCA. (**A**). Pedigree of the family with SCA7. (**B**). Pedigree of the family with SCA2. (**C**). Pedigree of the family with SCAR32. Squares indicate males, circles females, and arrows probands. Filled symbols indicate affected individuals; * relatives with molecularly confirmed cases of SCA (SCA2, SCA7, and SCAR32). Roman numerals I, II, III, IV, and V represent the generations in the pedigrees. (**A**,**B**) Autosomal dominant inheritance; (**C**) Autosomal recessive inheritance.

A 60-year-old female presented with gait problems and a family history of three daughters who also reported similar symptoms (Figure 1A).

She observed that she was veering to one side while walking and that her eyesight was deteriorating. These symptoms persisted until she sought care at our hospital’s neurology and ophthalmology clinic. Neurological examination revealed dysarthria, dysdiadochokinesis, dysmetria, increased deep tendon reflexes, spastic wide-swinging gait with a tendency to lateralize, and lack of coordination in lower limbs. A thorough examination by a clinical geneticist did not reveal other abnormalities or dysmorphias.

**Figure 2:**
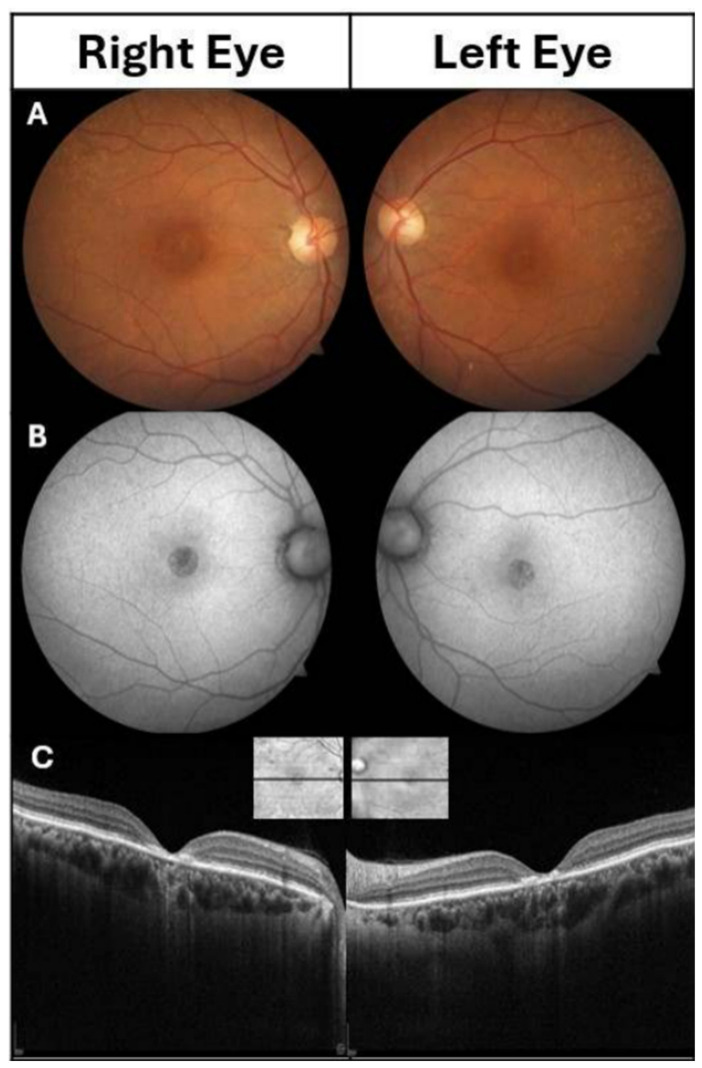
(**A**): Color fundus photo shows parafoveal RPE change with foveal sparing (“bull’s-eye”). (**B**): Autofluorescence shows central hypoautofluorescence with a surrounding hyperautofluorescent ring. (**C**): Spectral domain optical coherence tomography shows outer retinal thinning and ellipsoid zone disruption.

A comprehensive ophthalmic evaluation revealed a best-corrected visual acuity (BCVA) of 20/160 in both eyes. Color vision testing using the Ishihara plates revealed marked dyschromatopsia, with recognition limited exclusively to the control plate. Ocular motility assessment evidenced abnormal smooth pursuit movements. The patient reported a prior history of bilateral YAG laser peripheral iridotomy. Slit-lamp biomicroscopy of the anterior segment confirmed the presence of patent iridotomies in both eyes. Gonioscopic examination revealed iridotrabecular contact in three quadrants, along with peripheral anterior synechiae. Intraocular pressure (IOP) was measured at 15 mmHg in both eyes by applanation tonometry. The optic nerve head appeared within normal limits bilaterally, with a cup-to-disk ratio (CDR) of 0.5 and no signs of glaucomatous neuropathy (Figure 2).

Posterior segment examination disclosed bilateral macular changes characterized by a “bull’s eye” pattern of pigmentary alteration, consistent with macular dystrophy. No signs of macular edema or lipid exudation were observed. Spectral Domain Optical Coherence Tomography (SD-OCT) revealed bilateral thinning of all subfoveal retinal layers, particularly involving the photoreceptor layer. Additionally, there was a severe thinning of the ganglion cell layer (GCL).

Brain magnetic resonance imaging (MRI) revealed only olivopontocerebellar degeneration, and genetic testing was recommended. The repeat expansion analysis of the *ATXN7* gene was performed with triplet repeat primed PCR (TP-PCR), and the analysis identified a heterozygous pathogenic expansion (41 ± 1 CAG repeats), confirming the diagnosis of SCA7. Allelic ranges were interpreted according to established criteria, with normal alleles defined as 7–27 CAG repeats and pathogenic expansions ≥ 37 repeats. (Table 1).

### 2.2. Case 2

**Figure 3:**
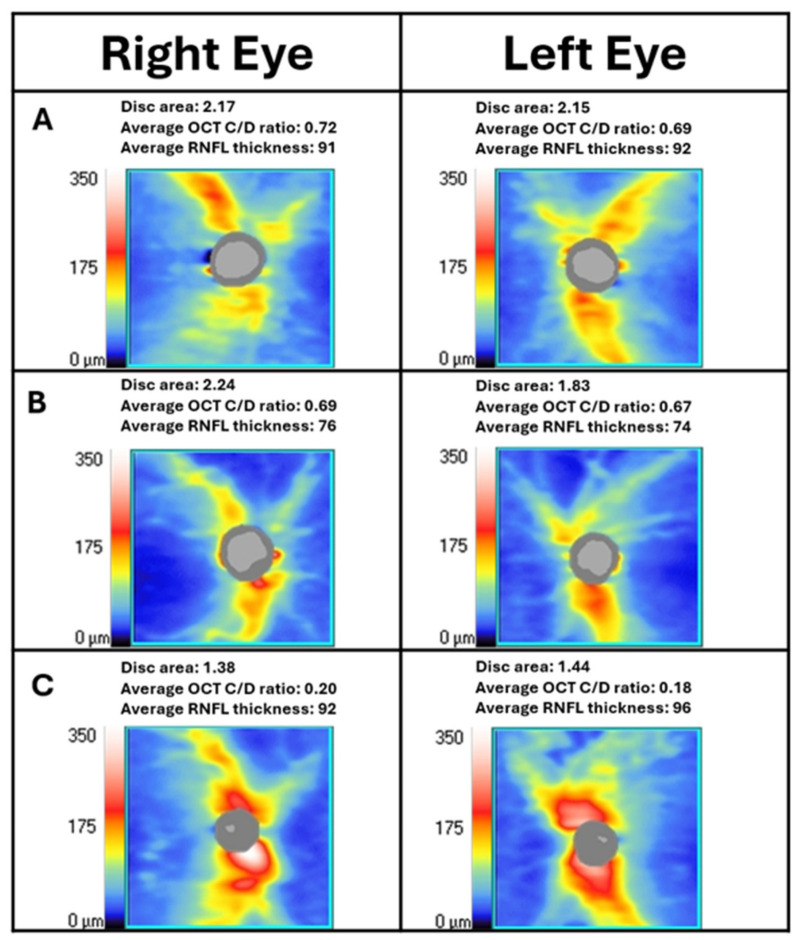
Optical coherence tomography–derived peripapillary retinal nerve fiber layer (RNFL) thickness maps of right and left eyes. Panels (**A**–**C**) show every case in the presented order, with disk area, average cup-to-disk ratio, and mean RNFL thickness.

**Figure 4:**
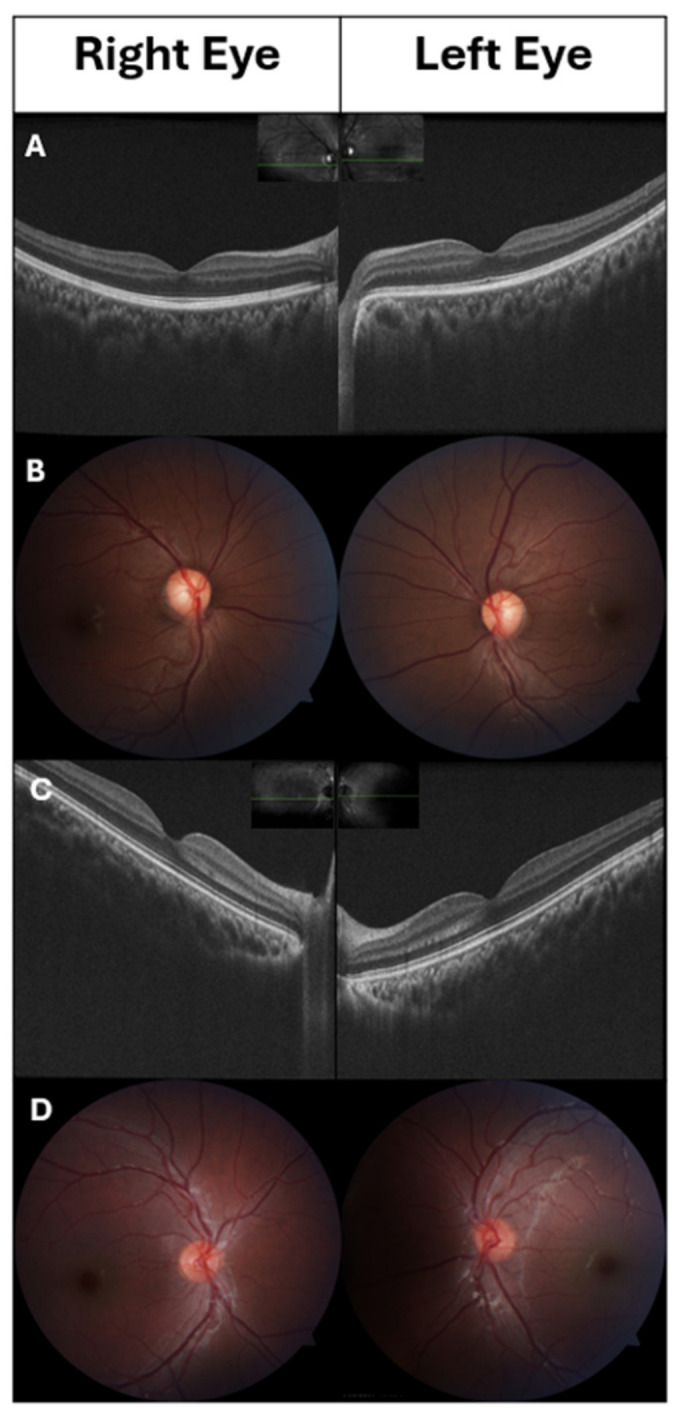
SD-OCT and color fundus photographs of cases 2 and 3. (**A**,**C**) Show the normal appearance of macular cross-section B-scans; (**B**,**D**) display optic disk and macular features.

A 24-year-old woman presented with a three-year history of progressive neurological symptoms, initially characterized by loss of coordination and balance. Over time, she developed dysarthria, difficulty manipulating objects, and dysphagia. She was evaluated by the neurology department, where a brain MRI was requested. The study revealed cerebellar atrophy, and she was subsequently referred to the ophthalmology and genetics department due to a strong paternal family history of ataxia (Figure 1B) (affecting her father, two paternal uncles, one paternal aunt, her grandmother, a great-aunt, and her great-grandmother). Ophthalmic examination revealed a BCVA of 20/50 in the right eye and 20/40 in the left eye, normal color vision, slow saccadic eye movements, preserved smooth pursuit eye movements, normal anterior segment, IOP of 14 mmHg in both eyes, normal macular morphology, and large CDR of 0.7. SD-OCT scans revealed a generalized thinning in the retinal nerve fiber layer (RNFL) and GCL (Figure 3) and a normal macular appearance (Figure 4).

In addition, her daughter, currently nine years old, began exhibiting symptoms at six years of age, including gait ataxia and dysarthria. Her brain MRI showed a generalized reduction in the cortico-subcortical cerebellar parenchyma with preserved global morphology. Given the clinical presentation and family history, targeted analysis of the CAG trinucleotide repeat expansion in the *ATXN2* gene was performed. Molecular testing identified a heterozygous pathogenic expansion of 51 CAG repeats, confirming the diagnosis of SCA2. Pathogenic alleles are generally defined by expansions exceeding 35 CAG repeats in the *ATXN2* gene. (Table 1).

### 2.3. Case 3

A 6-year-old male was evaluated in the pediatric neurology clinic due to gait difficulties. Without relevant family history, he began walking at 14 months of age and, since then, had required multiple consultations at various hospitals. Neurological examination revealed bradyphasia, dysarthria, and an ataxic gait. At ophthalmic evaluation, BCVA was 20/25 in both eyes. The patient presented horizontal nystagmus with otherwise normal gaze movements. Anterior segment examination was normal; the macula appeared normal (Figure 4), but optic disks were small with a small CDR (Figure 3). Brain MRI demonstrated reduced cerebellar volume, and whole-genome sequencing identified two heterozygous variants in the *PRDX3* gene, *PRDX3*(NM_006793.4):c.28C>T(p.Arg10)*, *PRDX3*(NM_006793.4):c.716C>T(p.Thr239Met), consistent with a diagnosis of SCAR32 (Figure 1C and Table 1).

## 3. Literature Review

A search was conducted in the OMIM database, reviewing each spinocerebellar ataxia listed under the platform’s phenotypic series of entries. The search was performed according to each mode of inheritance, including autosomal dominant, autosomal recessive, and X-linked forms, resulting in a list of the 84 registered types of spinocerebellar ataxias.

Subsequently, each type was individually reviewed in OMIM to collect information regarding the associated gene and protein, mode of inheritance, type of pathogenic variant, and the general clinical manifestations, neurological, and ophthalmological manifestations reported for each spinocerebellar ataxia.

**Figure 5:**
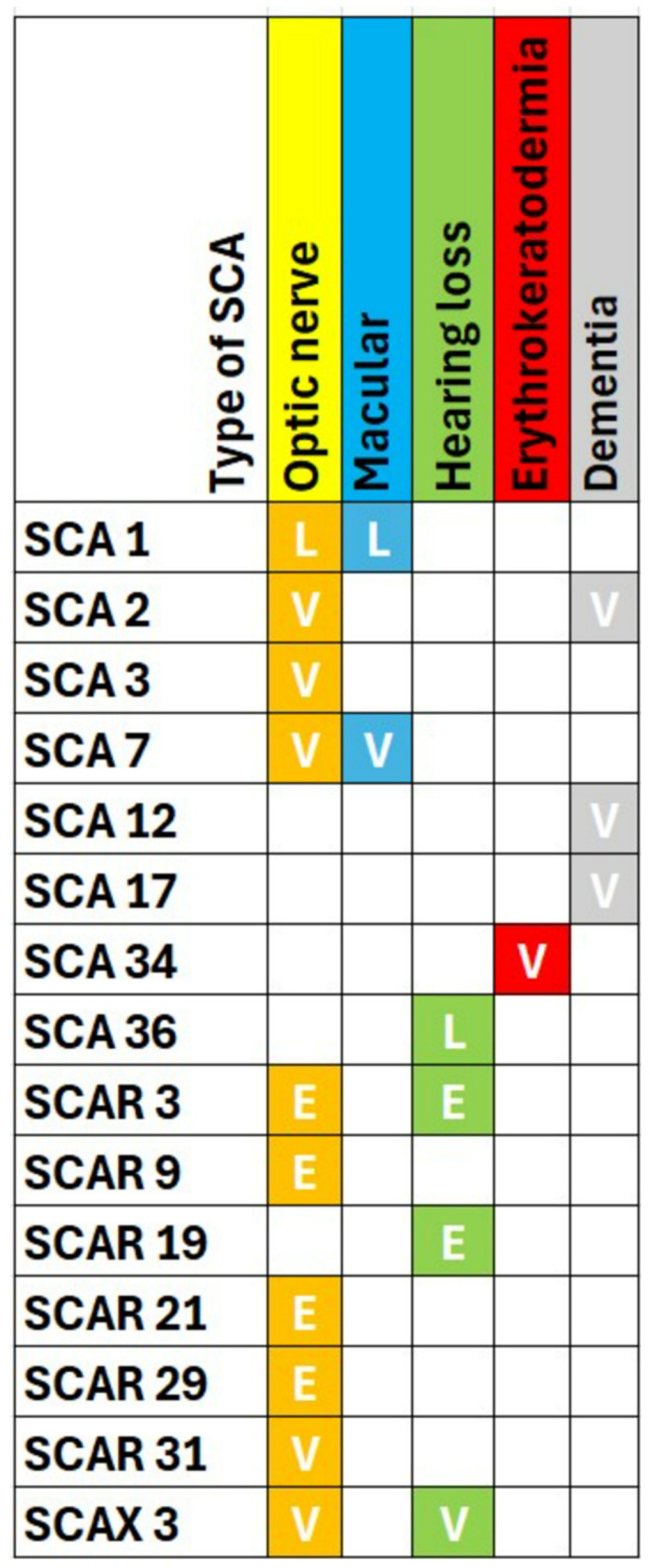
The image depicts the different types of SCAs with the key clinical features that differentiate them. Inside each box, the letter represents the age of onset; “E” denotes an early presentation, “L” a late presentation, and “V” a variable presentation.

Common manifestations shared by most spinocerebellar ataxias were identified, including movement disorders, gait abnormalities, nystagmus, and other recurrent neurological features. The analysis also highlighted clinical features that were not commonly shared among most spinocerebellar ataxias, including optic nerve involvement, macular involvement, hearing loss, erythrokeratoderma, and dementia. Accordingly, the spinocerebellar ataxias presenting these distinctive clinical characteristics were summarized in a figure (Figure 5). We have identified these five key clinical features that, in addition to the age of presentation, might help narrow the differential diagnosis.

Spinocerebellar ataxias with fewer than three reported cases were excluded, as the limited number of published cases did not provide sufficiently reliable information regarding their clinical manifestations.

When the information available in OMIM was insufficient, it was supplemented through a search of case reports in PubMed using the search term “spinocerebellar ataxia X” (where X corresponds to the ataxia number). The literature search included studies published up to October 2025.

The collected data were organized into a supplementary table. A total of 84 types of SCA have been reported; depending on the type of inheritance, 46 are AD, 32 are AR, and 6 are X-linked (Supplementary Material S1).

Macular anomalies are present in SCA1 and SCA7. In SCA1, the macular involvement is more subtle and silent. In a cohort of 20 patients with SCA1, 30% showed detectable macular anomalies by SD-OCT or ERG [6,7,8] and, in some cases, developed maculopathy before ataxia [8,9]. However, macular involvement is much more recognized and studied in SCA7, which is one of the most common forms of SCA, characterized by vision loss and ataxia. The cone-rod dystrophy is a characteristic sign in SCA7, and its appearance depends on the onset of the disease. In early onset, maculopathy is the first symptom, but in late onset, it appears simultaneously with the other symptoms [10,11].

Optic nerve involvement is present in SCA1, 2, 3, 7, SCAR3, 9, 21, 29, 31 and SCAX3. This is the key clinical feature that is shared by more types of SCA; however, it varies from a mildly enlarged cup-disk ratio to a severe optic atrophy [10,11,12,13,14]. In SCA2, an increased cup-disk ratio [8,10] has been reported as a characteristic feature, while in all other SCAs, optic nerve pallor is the common feature. In SCA1, 2, 3, and 7, retinal nerve fiber layer (RNFL) thinning has been reported [15]. SCAR9 differs from the rest of SCAs with optic nerve involvement because the optic atrophy is secondary to papilledema [16].

Hearing loss is present in SCA36, SCAR3, 19, and SCAX3. This is an important feature that tends to be severe and is not typically associated with tinnitus or vertigo. The penetrance of hearing loss among patients with these types of SCA varies from incomplete penetrance in SCA36 (74–88%) [16,17] and SCAR19 (75%) [18] to complete penetrance in SCAR3 [19,20] and SCAX3 [21].

Erythrokeratodermia is present only in SCA34, and it is characterized by symmetrical and progressive reddish papulosquamous lesions that become more intense during cold periods. They tend to remit by the age of 20–25 years but in some cases can persist [21,22]. In some cases, the erythrokeratodermia was present many years before the onset of the neurological symptoms [22,23].

Dementia is present in SCA2, 12, and 17, while other types of cognitive impairment not fulfilling the diagnosis of dementia have been reported in SCA1, 3, 6, 7, 8, 10, 21, 32, 48 [8,24,25], and SCAR8, 9, 10, 16, and 17. Intellectual disability is a characteristic feature of SCA 13 [26]. The severity of cognitive impairment-dementia varies significantly even in the same type of SCA; for instance, in SCA2, only 25% of patients fulfill the diagnosis of dementia [27], while the remaining present only with mild cognitive impairment and dysfunction of verbal memory [28]. In other types of SCA, dementia is more common, like in SCA17, in which dementia is a cardinal sign present in 90% of patients [29]. The age of presentation of cognitive impairment tends to be late in SCA2 and 12, but in SCA17, it can be as early as the second decade of life [30].

## 4. Discussion

An early referral to an ophthalmologist in the present case series shortened the differential diagnosis to different types of SCAs, which facilitated a faster molecular diagnosis. When evaluating a patient with a possible SCA, the prompt identification of the accompanying key clinical features greatly reduces the number of potential forms of ataxia and facilitates the selection of a molecular test specific to a single type or group of SCA. Differentiating the specific type of SCA from other ataxias facilitates the identification of the underlying cause, prevents delayed diagnosis of accompanying clinical symptoms, and enables a future personalized therapeutic approach. Additionally, it reduces diagnostic time, improves genetic counseling, minimizes unnecessary testing, and benefits both the patient and the healthcare system.

The differential diagnosis of ataxia is broad and should utilize targeted oculomotor, proprioceptive/vestibular bedside testing, and brain MRI to differentiate between true cerebellar syndromes and mimics, such as sensory ataxia from large-fiber neuropathy or dorsal column disease, bilateral vestibulopathy, and higher-level gait disorders. Among cerebellar ataxias, acquired causes can be classified by type of onset: acute (stroke, intoxication), subacute (post-infectious or immune/paraneoplastic), and chronic (toxic–metabolic/endocrine–nutritional, demyelinating, prion, structural). When the initial work-up is unrevealing, especially in progressive adult-onset cases, genetic etiologies should be pursued (AD SCAs, AR forms, such as Friedreich ataxia, or ataxia with oculomotor apraxia, and fragile X tremor/ataxia syndrome) using a tiered approach that includes repeat-expansion assays or genetic panels guided by age at onset, family history, and phenotype or whole-genome sequencing [31,32].

All patients with SCA typically present with one or all of the following four types of ocular movement abnormalities: saccadic dysmetria, slowed saccades, gaze-evoked nystagmus, and ophthalmoplegia [11,12,13,14,18,20,25,32,33,34,35,36,37,38,39,40,41,42,43,44,45,46,47,48,49,50,51,52,53,54,55,56,57,58,59,60,61,62,63,64,65,66,67,68,69,70,71,72,73,74,75,76,77,78,79,80,81,82,83,84,85,86,87,88,89,90,91,92,93,94,95,96,97]. All these abnormalities are related to the degeneration of the cerebellum and brainstem due to progressive neuronal degeneration [1,11,12,13,14,18,20,25,32,34,35,36,37,38,39,40,41,42,43,44,45,46,47,48,49,50,51,52,53,54,55,56,57,58,59,60,61,62,63,64,65,66,67,68,69,70,71,72,73,74,75,76,77,78,79,80,81,82,83,84,85,86,87,88,89,90,91,92,93,94,95,96,97,98,99,100,101]. In the cerebellum, the accumulation of proteins with extended polyglutamine causes degeneration of Purkinje cells, resulting in motor coordination loss and cerebellar atrophy [1,8,11,16,17,25,29,32,34,35,36,37,66]. And in the brainstem, the accumulation of polyglutamine proteins causes neuronal loss in areas such as the olivary and pontine nuclei, leading to neuronal death and dysfunction in both motor and sensory pathways [46,49,52,55,58,61,102].

Despite the common features shared by all SCAs, different key clinical features are present in only some types of SCAs due to their diverse genetic origins. The pathogenesis of some key clinical features has been partially explained. The macular and optic nerve involvement is caused by the accumulation of proteins with expanded polyglutamine tracts, primarily in photoreceptor cells. The accumulation of proteins leads to retinal degeneration due to disruptions in calcium homeostasis and oxidative stress, as well as optic nerve atrophy due to ganglion cell impairment. The pathogenesis of the other key clinical features varies depending on the type of SCA. Hearing loss results from the accumulation of repeated nucleotides, leading to neuronal degeneration and subsequent atrophy of both central and peripheral auditory pathways [103]. Erythrokeratodermia is caused by mutations in the *ELOVL4* gene, which is expressed in the epidermis and is involved in the elongation of fatty acids. The deficiency of these lipids impairs keratinocyte maturation and differentiation [104]. Cognitive impairment and dementia arise from progressive neuronal degeneration due to the accumulation of mutant proteins, which damage cells in the cerebellum and cerebello-cortical circuits, ultimately leading to atrophy and affecting executive functions, memory, and affectivity [56,105].

Despite the significant progress in molecular diagnostic techniques, the diagnosis of SCA still varies depending on the type, and the diagnostic alternatives include triplet-repeat expansion testing, gene panels, exome, or whole-genome sequencing (long reads). While there is no single genetic test capable of diagnosing all types of hereditary ataxias, it is still essential to narrow down the possible subtypes through early referral to a multidisciplinary clinical evaluation. The present case report highlights the benefits of early referral to an ophthalmologist, which facilitated a timely diagnosis in conjunction with a neurologist and a clinical geneticist.

The benefits of using fundus images to facilitate the diagnosis of systemic diseases, such as dementia [106], or cardiovascular disease [107] have been highlighted recently with the concept of oculomics [108]. Fundus photographs or OCT scans analyzed with artificial intelligence can detect subclinical microvascular alterations, retinal nerve fiber layer and ganglion cell complex thinning, and other imaging-derived biomarkers indicative of systemic vascular and neurodegenerative processes, often preceding clinical manifestations. The evaluation of the retina and fundus to identify biomarkers of systemic disease is gaining increasing relevance due to the integration of large databases with globally representative images and the growing progress in the field of medical artificial intelligence [108,109]. These new biomarkers can help in the early detection of pathologies such as Alzheimer’s and Parkinson’s disease [102], even before clinical manifestations appear, and might help in the differential diagnosis of rarer or more complex conditions.

In conclusion, a clinician facing a patient with ataxia should consider an early multidisciplinary evaluation, including ophthalmology, neurology, and clinical genetics, to identify key clinical features that narrow the differential diagnosis. Although the different types of SCA exhibit clearly overlapping neurological phenotypes, the presence of macular abnormalities (SCA1 and SCA7) or optic nerve involvement (SCA1, SCA2, SCA3, SCA7, SCAR3, SCAR9, SCAR21, SCAR29, SCAR31, and SCAX3) should guide the diagnostic work-up, optimizing resources for the management of the patient. A shorter list of differential diagnoses will facilitate the judicious selection of more tailored diagnostic tests, reduce diagnostic time, and prompt genetic counseling about the disease.

## Figures and Tables

**Table 1:** Clinical, ophthalmologic, neuroimaging, and molecular characteristics of the three patients with SCAs.

	Case 1 (SCA7)	Case 2 (SCA2)	Case 3 (SCAR32)
Age at symptom onset	50 years old	21 years old	14 months of age
Diagnostic delay	10 years	3 years	4.8 years
Family history	Yes	Yes	No
Neurological findings	Dysarthria, dysdiadochokinesis, dysmetria, increased deep tendon reflexes, spastic wide-swinging gait, lack of coordination in lower limbs.	Loss of coordination and balance, ataxic gait, dysarthria, difficulty manipulating objects, dysphagia	Bradyphasia, dysarthria, ataxic gait
MRI findings	Olivopontocerebellar degeneration	Cerebellar atrophy	Reduced cerebellar volume
Visual acuity	20/160 both eyes	RE:20/50 LE:20/40	20/25 both eyes
Ocular motility findings	Abnormal smooth pursuit movements	Slow saccadic eye movements	Horizontal nystagmus
Fundus/OCT findings	Color fundus photo shows parafoveal RPE change with foveal sparing (“bull’s-eye”). SD-OCT shows outer retinal thinning and ellipsoid zone disruption.	Generalized thinning in retinal nerve fiber layer and ganglion cell layer.	Optic disks were small with small cup-to-disk ratio
Genetic test performed	Triplet repeat primed PCR of CAG repeats in the *ATXN7* gene.	Analysis of CAG trinucleotide repeat expansion in the *ATXN2* gene	Whole-genome sequencing
Molecular result	Heterozygous pathogenic expansion 41 ± 1 CAG repeats in the *ATXN7* gene.	Heterozygous pathogenic expansion 51 CAG repeats in the *ATXN2* gene.	Two heterozygous variants *PRDX3*:c.28C>T(p.Arg10*) *PRDX3*:c.716C>T(p.Thr239Met)

## Data Availability

The original contributions presented in this study are included in the article/Supplementary Material. Further inquiries can be directed to the corresponding author.

## Supplementary Materials

The following supporting information can be downloaded at: https://www.mdpi.com/article/10.3390/brainsci16070756/s1. We organized the different types of SCAs that exist. We did not include SCAs that only had a single isolated case report. The material was organized by listing the OMIM registration number, the protein affected in each type, the inheritance pattern, the type of variant, and the main neurological and ophthalmologic manifestations.
